# Mapping Long COVID: Spatial and Social Inequities Across the United States

**DOI:** 10.1101/2025.08.21.25334183

**Published:** 2025-08-26

**Authors:** Zhetao Chen, Bingnan Li, Yewen Chen, Jialing Liu, Fangzhi Luo, Kehinde Olawale Ogunyemi, Yang Ge, Yuan Ke, Yang Yang, Xianyan Chen, Ye Shen

**Affiliations:** 1 Epidemiology & Biostatistics, College of Public Health, University of Georgia, Athens, GA, USA; 2 Department of Statistics, Franklin College of Arts and Science, University of Georgia, Athens, GA, USA

**Keywords:** Public health, long COVID, spatiotemporal disparities, spatial and social inequities

## Abstract

**Background:**

Long COVID affects a substantial portion of the U.S. population, yet its spatiotemporal distribution remains poorly characterized. The emergence of the Omicron variant and persistent sociodemographic disparities may contribute to regional variation in long COVID risk. Understanding the patterns of long COVID is essential to implementing targeted and equitable public health interventions.

**Methods:**

This retrospective study utilized data from the National COVID Cohort Collaborative (N3C), covering 5,652,474 COVID-19 cases and 41,694 long COVID cases across 1,063 U.S. counties from 2021 to 2024. Temporal patterns of long COVID were analyzed before and after the Omicron variant’s emergence, and spatial patterns were assessed using Moran’s I and Getis statistics. Bayesian spatial random effect models were employed to evaluate the associations between long COVID incidence and sociodemographic factors such as economic vulnerability, healthcare access, and mobility.

**Findings:**

Quarterly long COVID incidence ranged from 0.015% to 14.29%. Before the emergence of the Omicron variant, incidence was 204 cases per 10,000 COVID-19 cases, compared with 248 cases per 10,000 COVID-19 cases after Omicron emergence (p < 0.001). After Omicron’s emergence, 48.8% [328 of 673] of counties showed significant spatial correlation (p < 0.05), up from 43.5% [293 of 673] prior. High-risk areas became more concentrated in inland regions, while low-risk areas clustered along the East Coast. Long COVID incidence was significantly associated with economic vulnerability, limited healthcare access, and mobility constraints, with these sociodemographic disparities consistently driving its spatial disparities over time.

**Interpretation:**

These findings underscore the need to address spatial and social inequities in long COVID risk. Targeted public health interventions, particularly in economically and geographically vulnerable regions, are essential to ensure equitable access to diagnosis, care, and resource allocation.

**Funding:**

Y. Shen received partial support from NIH grants/contracts R35GM146612, R01AI170116, and 75N93019C00052.

## 1. Introduction

Long COVID, or post-acute sequelae of SARS-CoV-2 infection (PASC), has become a major public health concern due to its long-term impacts^1,2^. In 2022, approximately 6.9% of adults reported having ever experienced long COVID, and 3.4% were currently experiencing it, illustrating the substantial prevalence and persistence of this condition^3^. These long-term symptoms can involve multiple physiological systems, such as respiratory, neurological, cardiovascular, and gastrointestinal, leading to fatigue, cognitive impairment, shortness of breath, and other chronic issues^4^. Although many epidemiological studies have explored individual-level risk factors for long COVID-19^5,6^, our understanding of its spatial and temporal incidence dynamics remains limited.

The distribution of long COVID exhibits complex spatial and temporal disparities. The spread of acute COVID-19 was highly uneven across regions^7^, and emerging evidence suggests that long COVID prevalence also varies geographically^8,9^. According to a June 2022 survey conducted by the Centers for Disease Control and Prevention (CDC), the prevalence of current long COVID symptoms among the United States (U.S.) adults varied substantially across states, ranging from 4.5% in Hawaii to 12.7% in Kentucky^10^. Additionally, temporal shifts have also been observed, particularly before and after the emergence of the Omicron variant^11^. Beyond these patterns, socioeconomic and demographic factors can play a crucial role in accounting for long COVID risk^10,12^. Prior studies have shown that prevalence differs across age groups and racial/ethnic populations^13^. However, previous studies have rarely examined the spatiotemporal patterns of long COVID incidence and how these factors dynamically influence spatial disparities in long COVID incidence.

In this study, we analyzed county-level long COVID data from the National COVID Cohort Collaborative (N3C) and sociodemographic data from the Social Vulnerability Index (SVI), covering 1,063 U.S. counties over the period 2021–2024. Our analysis advances prior work by providing a detailed spatiotemporal assessment and evaluating the influence of community-level social factors on incidence patterns. These findings can help guide targeted public health strategies and inform equitable healthcare resource allocation.

## 2. Methods

### 2.1 Study population and data collection

We constructed two datasets from the N3C database (version 185), one for acute COVID-19 and one for long COVID, based on daily EHR data from July 1, 2020 to March 31, 2024 (Figure S1 of the Supplementary Material). The data included individual-level records of confirmed cases, ZIP codes of site locations, and associated demographics, contributed by 62 sites across U.S. counties. Due to the sparse spatial distribution of sites, this study focuses on county-level analysis rather than site-level analysis to improve the robustness of the results. By summary, 1,063 of 3,144 counties with at least one reported long COVID case were included in the incidence analysis. Moreover, to enable valid pre-post comparisons around the Omicron-dominant period, we further restricted the related comparison analysis to 673 counties with available long COVID data both before and after this period.

To understand factors potentially influencing long COVID incidence, we incorporated socioeconomic and demographic variables from SVI, developed by the Centers for Disease Control and Prevention and the Agency for Toxic Substances and Disease Registry^14^. We used two versions of the SVI data to align with our pre- and post-Omicron analyses: the 2022 SVI, based on the 5-year (2018–2022) American Community Survey (ACS), was used for the post-Omicron period, and the 2020 SVI, based on the 5-year (2016–2020) ACS, was used for the pre-Omicron period. This approach ensures that the socioeconomic context used in each analysis reflects the corresponding time frame as accurately as possible, which is particularly important given potential shifts in community characteristics over the course of the pandemic. SVI was designed to identify communities that may need support before, during, or after public health emergencies. In this study, nine SVI-related variables, along with nine demographic and geographic factors, were analyzed. In particular, these SVI variables reflected socioeconomic status, household characteristics, racial and ethnic minority status, housing type, and access to transportation^15^.

### 2.2 Study outcomes

The incidence of long COVID was used as the outcome of interest. To assess evolution of long COVID incidences over time, we employed dynamic incidence risk calculation method (Figures S2–S3 of the Supplementary Material). By comparing long COVID EHR cases on specific time periods across various spatial regions with the corresponding at-risk acute COVID-19 populations, we derived two outcomes for the long COVID incidence. One was the incidence before and after the Omicron-dominant period (i.e., January 2022), which was set based on the standard provided by the WHO^16^. The other outcome was the quarterly incidence computed using three-month intervals based on natural calendar months given emerging evidence on the seasonal variability of COVID-19^17^.

### 2.3 Statistical analysis

To assess spatial local patterns in long COVID incidence across U.S. counties, we employed both local Moran’s I and Getis statistics^18,19^ (Figure S4 of the Supplementary Material). Local Moran’s I quantifies the degree of similarity or dissimilarity between a county and its neighbors, with positive values indicating similarity and negative values indicating dissimilarity. In contrast, Getis identifies statistically significant spatial clusters of high (hot spots) or low (cold spots) incidence.

To accurately estimate potential relationships between variables and long COVID incidence, simple stepwise regression methods were used to identify a subset of variables that best explained the variation in long COVID incidence. Given the right-skewed distribution of long COVID incidence, we applied a logarithmic transformation to stabilize variance and improve model performance (Figure S8 of the Supplementary Material). Based on the selected variables, spatial random effects models were then employed to account for spatial heterogeneity. The model can be written as:

$$y_{i}=X_{i}\beta +W_{i\epsilon S_{g}}+\epsilon_{i},$$

where *y*_*i*_ is the incidence of the *i*-th county, ***X*** is the covariables selected via stepwise regression models. To avoid confounding introduced by spatially correlated random effects, we modeled the zero-mean Gaussian spatial random effect $W_{i\epsilon S_{g}}$ as subregion-level independent errors, with variances $\tau_{g}^{2}$ varying across subregions for *g* = 1, ⋯, 5(Figure S5 of the Supplementary Material).

The term *ϵ*_*i*_ represents zero-mean Gaussian errors with the variance of *τ*^2^. To avoid potential multicollinearity among covariables, under a Bayesian setting, we used a ridge-like prior for the coefficients ***β*** to stabilize estimates, specified as zero-mean Gaussian with a small variance of 0.1. All analyses were conducted using R statistical software (version 4.4.0)^20^.

## 3. Results

### 3.1 Overall description

**Figure 1.**
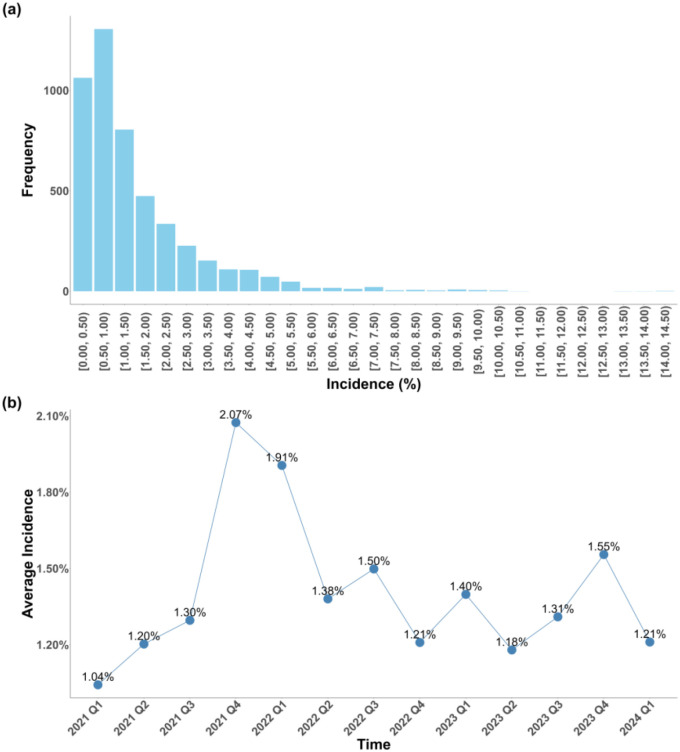
Long COVID incidences. (a) Its distribution in each interval; and (b) Time-trend of average incidence across counties from January 2021 to March 2024.

From 2020 to 2024, a total of 5,652,474 COVID-19 cases were recorded in the N3C database, among which 41,694 long COVID cases were identified based on the dynamic incidence risk described in Figure S2, resulting in an overall incidence risk of 0.74% across the study period. The quarterly incidence of long COVID ranged from 0.015% to 14.29%, with 66.0% of counties recording incidence rates in the interval [0.015%, 1.50%] (Figure 1a). The average incidence across counties peaked in the fourth quarter of 2021 (October-December) at 2.07%, followed by another high in the first quarter of 2022 (January-March) at 1.91%, before declining thereafter (Figure 1b). These two peaks coincided with the period when the Omicron variant rapidly replaced Delta as the dominant strain in the U.S. during 2022 Q1. A Wilcoxon signed-rank test (p < 0.0001) indicated a statistically significant difference in long COVID incidence before and after the Omicron-dominant period, suggesting a shift in the trend associated with the emergence of Omicron.

To facilitate geographic comparisons, we adopted a commonly used five-region classification of the U.S.^21^(Figure S5). All five U.S. regions exhibited a substantial increase in long COVID incidence following the Omicron surge (Table S1 of the Supplementary Material). Specifically, more than two-thirds of counties in each region experienced an increase with the Northeast showing the highest proportion (75%) and the South showing the lowest (66.67%). Despite regional variation, the overall proportion of counties with increased incidence was moderately high at 68.95%, highlighting the significant epidemiologic shift associated with the emergence of Omicron. In detail, the patterns of increasing or decreasing county-level incidence before and after the Omicron-dominant period exhibited local spatial similarity (Figure S6 of the Supplementary Material). Moreover, the incidence varied notably across regions (Table S2 of the Supplementary Material). The West consistently exhibited the highest average incidence, increasing from 2.42% before Omicron to 2.86% after its emergence. The Midwest and Northeast also experienced considerable increases, with post-Omicron incidences reaching 2.79% and 2.45%, respectively. Interestingly, while the South maintained a moderate level of incidence, increasing from 2.21% to 2.53%, the Southwest was the only region where the average incidence slightly declined after the Omicron surge, from 1.85% to 1.78%.

### 3.2 Spatial correlation analysis

Local Moran’s I analysis revealed that the total proportion of counties with significant correlations increased from 43.5% before the emergence of Omicron to 48.8% afterward (Table S3 of the Supplementary Material). Positive spatial autocorrelation was more prevalent than negative in both periods. Specifically, the proportion of counties showing positive correlations rose from 32.7% to 35.4%, while the proportion with negative correlations increased from 10.8% to 13.4%. Regionally, the proportion of counties with positive correlations decreased in the Northeast (from 43.3% to 21.7%) and South (from 51.6% to 50.8%), remained relatively stable in the Midwest (from 22.8% to 23.2%), and increased notably in the West (from 11.1% to 32.2%) and Southwest (from 0.0% to 38.2%). For negative correlations, the proportion declined slightly in the South (from 14.7% to 14.3%), Northeast (from 8.3% to 5.0%), and Southwest (from 11.8% to 8.8%), but increased in the Midwest (from 7.2% to 12.7%) and West (from 11.1% to 20.0%). Overall, counties with significant spatial correlations were primarily located in the Middle and eastern regions (Figure S7 of the Supplementary Material).

### 3.3 High- and low-risk areas

**Figure 2.**
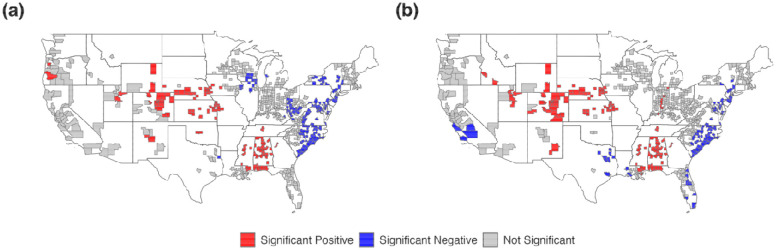
Spatial clustering of long COVID incidence across U.S. counties based on Getis statistic before (a) and after (b) Omicron dominance.

Getis statistics identified notable shifts in the spatial distribution of both high-risk and low-risk areas for long COVID incidence before and after the dominance of the Omicron variant (Table 1). The total proportion of counties classified as high-risk increased markedly from 14.9% to 27.2%. Regionally, the Midwest experienced the largest increase in high-risk counties, rising from 16.9% to 35.9%, followed by the West (22.2% to 40.0%) and the South (14.7% to 23.8%). In contrast, the Northeast had no high-risk counties identified in either period, and the Southwest showed a slight decrease from 8.8% to 5.9%. Conversely, the total proportion of counties identified as low-risk declined from 28.7% to 21.5%. The South and Northeast showed decreases in low-risk counties – from 51.6% to 41.3% and 51.7% to 26.7%, respectively. The Midwest saw a complete drop from 13.1% to 0.0%, while the West increased slightly from 0.0% to 12.2%. Notably, the Southwest showed a substantial rise in low-risk counties, from 2.9% to 41.2%. Overall, the results indicate an expansion of high-risk clusters – especially across the Midwest and West – after the emergence of the Omicron variant, whereas low-risk areas became more concentrated in the Southwest, as visualized in Figure 2.

### 3.4 Associations between social vulnerability factors and long COVID incidence

**Figure 3.**
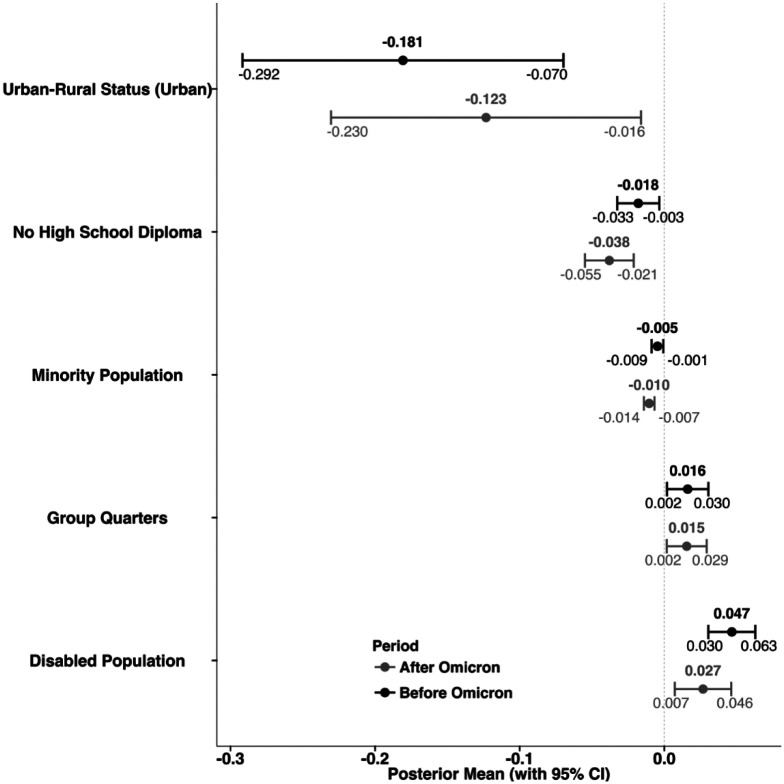
Posterior estimates (95% credible intervals (CIs)) of regression coefficients that measure the relationships between social vulnerability factors and long COVID incidence based on Bayesian spatial random effect models.

Stepwise regression identified different sets of variables for the periods before and after Omicron dominance (Table S4 and S5 of the Supplementary Material). The spatial random effect models were then developed using the selected variables. Figure 3 presents the results for variables that were selected in both periods, while variables that differed between the two periods are shown in Figure S10 of the Supplementary Material. Urban counties consistently exhibited lower long COVID incidence than rural counties in both the pre- and post-Omicron periods (−0.181, 95% CI: −0.292 to −0.070 before; −0.123, −0.230 to −0.016 after). Counties with a higher proportion of adults without a high school diploma also showed significant negative associations across both periods (−0.018, −0.033 to −0.003 before; −0.038, −0.055 to −0.021 after), as did those with a higher proportion of racial and ethnic minority populations (−0.005, −0.009 to −0.001 before; −0.010, −0.014 to −0.007 after). In contrast, a higher proportion of disabled residents was significantly positively associated with long COVID incidence only before Omicron (0.047, 0.030 to 0.063), with a weaker but still significant association observed afterward (0.027, 0.007 to 0.046).

Similarly, counties with more residents living in group quarters showed a significant positive association in both periods (0.016, 0.002 to 0.030 before; 0.015, 0.002 to 0.029 after). Additional variables that were significantly associated with long COVID incidence but only in one period are shown in Figure S10. For example, the proportion of the population aged over 65 (−0.021, −0.034 to −0.008), multi-unit housing (−0.013, −0.022 to −0.005), limited English proficiency (0.040, 0.009 to 0.072), and the proportion below 150% of the federal poverty level (0.017, 0.006 to 0.027) were significant only after Omicron, whereas first vaccination rate (−0.006, −0.009 to −0.002) and housing cost burden (0.015, 0.003 to 0.028) were significant only before Omicron.

In particular, the spatial random effect methods improved model fit and strengthened the robustness of statistical inference based on histograms of residuals, along with the results of Anderson–Darling test (Figure S9 of the Supplementary Material).

### 3.5 Impacts of sociodemographic disparities on the long COVID incidence

**Figure 4.**
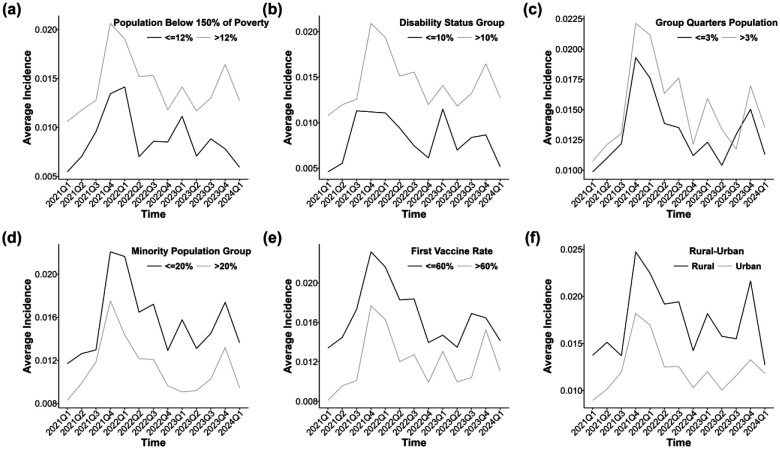
Temporal trends in long COVID incidence across different levels of six social vulnerability factors: (a) poverty status (population below 150% of the federal poverty level), (b) disability prevalence, (c) residence in group quarters, (d) racial and ethnic minority composition, (e) COVID-19 vaccination coverage, and (f) rural – urban classification.

Figure 4 (a–f) presents the temporal trends in long COVID incidence stratified by selected SVI-related factors, with patterns broadly consistent with the findings of spatial random effect models. Counties with a higher proportion of residents living below 150% of the poverty level (>12%) consistently exhibited higher long COVID incidence than those with lower poverty levels (≤12%) (Figure 4a). Similarly, counties with a greater proportion of disabled residents (>10%) experienced higher incidence compared to those with lower disability prevalence (≤10%) across most quarters (Figure 4b). In Figure 4c, counties with more residents living in group quarters (>3%) also showed elevated incidence relative to those with ≤3%. Interestingly, Figure 4d indicates that counties with higher minority population proportions (>20%) tended to have lower long COVID incidence than those with lower proportions (≤20%), particularly after 2022. Counties with higher vaccination rates (>60%) consistently reported lower incidence than those with lower rates (≤60%) (Figure 4e). Finally, rural counties exhibited higher long COVID incidence than urban counties throughout the study period, with a marked divergence following the emergence of Omicron (Figure 4f). In particular, the threshold for each variable was selected based on comparison analysis to ensure that, under the given threshold, the difference in long COVID incidence related to the variable was statistically significant in most of the six subregions (Table S6 of the Supplementary Material). This approach ensured the robustness of the results above.

## 4. Discussion

This study offers a comprehensive view of long COVID incidence by analyzing its spatiotemporal patterns and associations with area-level socioeconomic and demographic factors, extending beyond traditional individual-level analyses. Using a large U.S. dataset of 5,652,474 COVID-19 and 41,694 long COVID cases from 2021 to 2024, we identified significant spatiotemporal differences in its epidemiology. Spatial clustering of incidence was observed across many counties (p < 0.05). Notably, low-incidence areas were more concentrated on the U.S. East Coast, while high-incidence areas distributed inland regions, likely reflecting pre-existing disparities in health (healthcare and public health) services coverage and available social infrastructure. This epidemiological pattern may in part be explained by evidence of socioeconomic and demographic clustering in these regions, where Americans of higher socioeconomic status have been reported to be migrating to the coastal regions while those with lower socioeconomic status are moving to the inland regions^22^. These findings further suggest that low-income and underserved populations in inland regions may face a disproportionately higher risk of developing long COVID. This elevated vulnerability is likely driven by the higher prevalence of contributing factors in these communities, such as comorbidities and smoking^23,24^.

This study identifies SVI factors associated with long COVID and clarifies their role in spatiotemporal disparities. Our analysis identified rurality, poverty levels, disability prevalence, institutional living environments, minority composition, vaccination coverage, and residential crowding as key factors influencing long COVID incidence, with varying effects across time periods. Specifically, we consistently observed higher long COVID incidence between 2021 and 2024 in counties characterized by rural status, higher disability prevalence, and a larger share of residents in institutional living environments. Counties with higher poverty levels and greater residential crowding became more strongly associated with higher long COVID incidence after the emergence of the Omicron variant. This finding highlights the disproportionate burden of long COVID among economically vulnerable communities. Existing healthcare and social determinants of health inequities likely played a critical role in shaping these patterns, as lower healthcare access and higher economic vulnerability contribute to prolonged post-acute symptoms and delayed medical diagnosis and treatment^25^. In contrast, higher COVID-19 vaccination coverage was associated with lower long COVID incidence primarily in the pre-Omicron period, suggesting a potential early protective effect against long-term complications. Interestingly, counties with higher minority populations and lower educational attainment showed lower reported incidence. This may reflect underdiagnosis and ecological bias, as county-level metrics may not reflect the characteristics of individuals in the N3C dataset. Moreover, individuals from minority backgrounds or with lower education levels may be less likely to seek care for persistent post-COVID symptoms due to health literacy challenges, language barriers, limited healthcare access, or financial and structural obstacles. As a result, these populations are more likely to be underrepresented in her-based long COVID case definitions. As such, future studies leveraging N3C data should interpret these associations with caution and consider the potential impact of ecological bias and data representativeness when evaluating racial and ethnic disparities or levels of educational attainment in long COVID burden.

Given the significant spatiotemporal disparities in long COVID incidence, policy responses should promote equitable diagnosis and care. Targeted healthcare resource allocation with risk stratification is essential, with high-incidence areas requiring specialized long COVID clinics, rehabilitation centers, and increased funding for chronic disease prevention and control, while low-incidence areas should be assessed for under-reporting, under-diagnosis, clinician competency, and population awareness gaps^26^. Given that many high-risk regions are in underserved Midwestern areas, expanding telemedicine services and deploying mobile health units can improve accessibility to specialized care for acute COVID-19, long COVID, and existing comorbidities^27^. Additionally, in areas with limited healthcare facilities and low population engagement with the healthcare system for long COVID diagnosis and care, outreach programs and social support initiatives should be implemented to improve early detection and intervention and reduce the entrenched individual-level and social determinants of health inequities. Related policies, such as the disability measure^28^, can also be adjusted to reflect geographic and demographic disparities, ensuring adequate support for long COVID patients facing economic and healthcare barriers^26^. Addressing these disparities through policy-driven health interventions, including healthcare accessibility improvements and targeted public health interventions implementation will help mitigate the short-term and long-term impact of long COVID, ultimately leading to better health outcomes for vulnerable populations, reduced associated health costs for the governments, and improved health equity.

This study has several limitations. First, due to constraints in the N3C EHR database, approximately 35% of long COVID records lacked corresponding COVID-19 EHR records, likely because many patients experienced mild symptoms and did not seek healthcare, leading to a dataset biased toward individuals with more severe acute infections^29^. Second, consistent with data from most surveillance systems, the long COVID incidence estimated from N3C sentinel surveillance system might not represent the true burden of the disease due to under-ascertainment bias from symptomatic cases who do not seek health care due to healthcare access (physical and/or economic) constraints and poor awareness of long COVID symptoms. Third, sampling bias may have been introduced due to the limited number of sentinel sites included in the database, and another important limitation is the uneven distribution of these sites across U.S. regions, which may have led to some areas being oversampled while others were under-sampled. This imbalance could introduce regional biases in the data, potentially affecting observed incidence rates and risk factor associations, thereby reducing the generalizability of the findings to the broader U.S. population, particularly in regions with fewer or no sentinel sites where healthcare access and demographic characteristics may differ significantly. Fourth, our method for estimating incidence risks involved excluding patients who had not received a long COVID diagnosis within 180 days after their COVID-19 infection, which may have led to measurement bias resulting from an underestimation of the actual disease burden. Moreover, it is also possible that the differences in the data collection time period for the N3C EHR and SVI databases have contributed to measurement bias. Fifth, due to uneven data collection in the N3C EHR system, regions with well-resourced healthcare facilities may report higher long COVID diagnoses due to improved detection from robust screening and diagnostic practices, while under-resourced areas may underreport cases, potentially distorting geographic patterns. Sixth, to maintain data stability, we excluded counties with fewer than 20 COVID-19 records, which may have further affected spatial representation. Finally, since long COVID is an extremely heterogeneous condition encompassing a wide range of symptoms^30^, our study did not differentiate cases by specific symptom profiles, which may obscure important subgroup trends. Future research should explore symptom-specific patterns and pre-existing medical conditions to enhance the understanding of long COVID incidence and its spatiotemporal patterns and wider social determinants of health for improved design and delivery of tailored and targeted health and social interventions at both the population and healthcare system levels.

## Supplementary Material

1

Supplementary Material provided more details, including descriptions of the datasets, data processing, and additional results.

## Figures and Tables

**Table 1.** Changes in the number and proportion of counties with high and low long COVID incidence risk before and after Omicron dominance across different subregions of the United States (673 counties).

Region	The number (proportion^a^) of counties in high risk		The number (proportion) of counties in low risk	
	Before	After	Before	After
South	37 (14.7%)	60 (23.8%)	130 (51.6%)	104 (41.3%)
Northeast	0 (0.0%)	0 (0.0%)	31 (51.7%)	16 (26.7%)
Midwest	40 (16.9%)	85 (35.9%)	31 (13.1%)	0 (0.0%)
West	20 (22.2%)	36 (40.0%)	0 (0.0%)	11 (12.2%)
Southwest	3 (8.8%)	2 (5.9%)	1 (2.9%)	14 (41.2%)
Total	100 (14.9%)	183 (27.2%)	193 (28.7%)	145 (21.5%)

a Proportion = (Number of high- or low-risk counties) / (Total number of counties in the region)

## Data Availability

All data used in this study is available through the N3C Enclave to approved users. See https://covid.cd2h.org/for-researchers for instructions on how to access the data. We used N3C data from version 185.
