# Supplementary material for: Mapping Long COVID: Spatial and Social Inequities Across the United States
Source: medRxiv. 2025 Aug 26:2025.08.21.25334183. Preprint. [Version 1] doi: 10.1101/2025.08.21.25334183 (PMC12407596; doi:10.1101/2025.08.21.25334183)
Supplement: 1 [file NIHPP2025.08.21.25334183V1-supplement-1.pdf]

medRxiv preprint doi: https://doi.org/10.1101/2025.08.21.25334183; this version posted August 26, 2025. The copyright holder for this preprint (which was not certified by peer review) is the author/funder, who has granted medRxiv a license to display the preprint in perpetuity.
It is made available under a CC-BY 4.0 International license .

Supplementary Material: Mapping Long COVID: Spatial and Social Inequities Across the United States

Zhetao Chen[1†], Bingnan Li[2†], Yewen Chen[1†], Jialing Liu[1], Fangzhi Luo[1], Kehinde Olawale Ogunyemi[1], Yang Ge[1], Yuan Ke[2], Yang Yang[2], Xianyan Chen[1*], Ye Shen[1*], on behalf of N3C consortium

Affiliations:
[1]Epidemiology & Biostatistics, College of Public Health, The University of Georgia, Athens, GA, USA
[2]Department of Statistics, Franklin College of Arts and Science, The University of Georgia, Athens, GA, USA

† The first three authors contribute equally to this article

* **Correspondence**:

X. Chen: xychen@uga.edu
Y. Shen: yeshen@uga.edu

medRxiv preprint doi: https://doi.org/10.1101/2025.08.21.25334183; this version posted August 26, 2025. The copyright holder for this preprint (which was not certified by peer review) is the author/funder, who has granted medRxiv a license to display the preprint in perpetuity.
It is made available under a CC-BY 4.0 International license .

S1 EHR of long COVID and data processing

Given the inherent characteristics of electronic health record (EHR) data, which primarily capture individuals with more severe symptoms who seek in-person medical care, our long COVID cohort is likely biased toward patients experiencing more severe or persistent symptoms. Notably, approximately 40% of identified long COVID cases lack corresponding acute COVID-19 records in the EHR, likely due to patients experiencing mild or asymptomatic acute infections and recovering at home without medical intervention, resulting in an absence of documented acute disease. As our objective is to estimate and compare the incidence risk of long COVID across regions, we define the at-risk population as individuals with a confirmed acute COVID-19 diagnosis, allowing for standardized denominator calculations. long COVID cases without a documented acute infection are therefore excluded to ensure the validity of risk estimation. This exclusion is essential for minimizing misclassification bias, as the absence of an acute COVID-19 record precludes accurate determination of symptom onset relative to infection, leading to potential inflation or deflation of risk estimates. Additionally, these cases may represent individuals who sought care only for persistent symptoms rather than those with a well-defined transition from acute infection to long COVID, introducing heterogeneity that could bias comparisons across regions. Furthermore, the completeness of acute COVID-19 documentation varies across healthcare systems, with differences in testing accessibility and healthcare-seeking behaviors influencing EHR records. By restricting the cohort to individuals with documented acute infections, we enhance the comparability of risk estimates across populations and mitigate potential confounding arising from regional variations in EHR capture. This approach ensures a robust and interpretable assessment of long COVID incidence following acute infection.

For identifying long COVID cases, we utilized ICD-10 codes B94.8 and U09.9 as diagnostic indicators. Since U09.9 was officially introduced as the long COVID diagnosis code on October 1, 2021, diagnoses related to long COVID prior to this date predominantly used the B94.8 code. Therefore, we applied a temporal cutoff on October 1, 2021, selecting EHR records with B94.8 diagnoses before this date and U09.9 diagnoses thereafter. This approach ensures comprehensive coverage of long COVID cases across different time periods, accounting for the evolution of diagnostic practices.

We initiated data cleaning using the original N3C dataset. Overall cleaning process was summarized in **Figure S1**. On one hand, COVID-19 patient records were cleaned, and on the other, long COVID patient records were similarly processed. Only records corresponding to a patient's first COVID-19 infection or first long COVID infection were retained. In the process of filtering N3C EHR data, we also applied site-level selection criteria. During data cleaning, we observed that certain sites had ceased their collaboration with N3C by the time long COVID cases began to emerge. Including EHR data from these sites could potentially bias our analysis by underestimating the incidence of long COVID, thereby affecting subsequent data interpretation. To ensure data quality and consistency, we excluded sites with a long COVID incidence rate below 0.1%, as we deemed these sites invalid for analysis due to insufficient case reporting or incomplete data coverage. This site-level filtering helps improve the robustness of our incidence estimates and ensures more reliable regional comparisons.

medRxiv preprint doi: https://doi.org/10.1101/2025.08.21.25334183; this version posted August 26, 2025. The copyright holder for this preprint (which was not certified by peer review) is the author/funder, who has granted medRxiv a license to display the preprint in perpetuity.
It is made available under a CC-BY 4.0 International license .

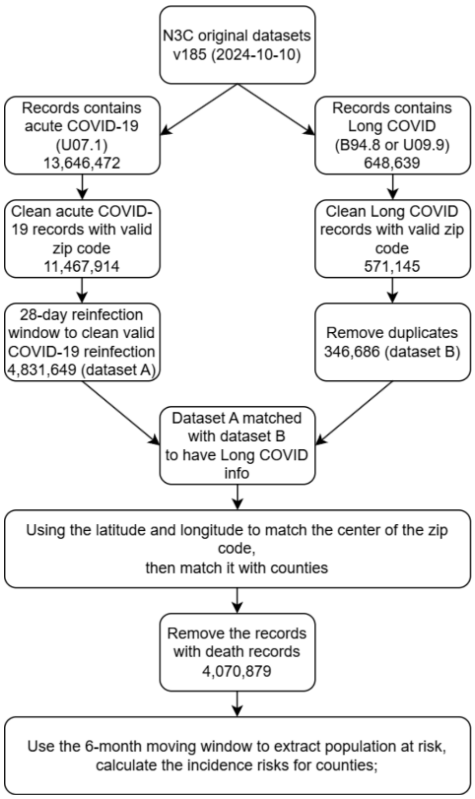

Figure S1: Diagram of data filtering and cleaning.

S2 Dynamic incidence risk and definition of long COVID incidence

It is important to emphasize that all long COVID incidence calculations in this study are based exclusively on individuals who were previously diagnosed with acute COVID-19. That is, the at-risk population for all incidence metrics consistently includes only those with documented acute COVID-19. For the dynamic incidence risk algorithm shown in **Figure S1**, the criteria for defining the dynamic population at risk are as follows:

(a) Earliest inclusion date for the population at risk: We determined the start of the population at risk as 180 days prior to the earliest date of the target period. For example, for long COVID cases identified between January 1, 2021, and March 31, 2021, the corresponding population at risk consists of COVID-19 patients recorded in the EHR starting from July 1, 2020.

(b) Latest inclusion date for the population at risk: Following CDC guidelines, we adopted a one-month timeframe as the allowable window between the COVID-19 diagnosis and the long COVID diagnosis. The study [Crowd-sourced machine learning prediction of long COVID using data from the National COVID Cohort Collaborative] suggests that even when using B94.8 and U09.9 codes, a more conservative approach can be applied by using a 28-day threshold as a filter to refine long COVID data.

medRxiv preprint doi: https://doi.org/10.1101/2025.08.21.25334183; this version posted August 26, 2025. The copyright holder for this preprint (which was not certified by peer review) is the author/funder, who has granted medRxiv a license to display the preprint in perpetuity. It is made available under a CC-BY 4.0 International license .

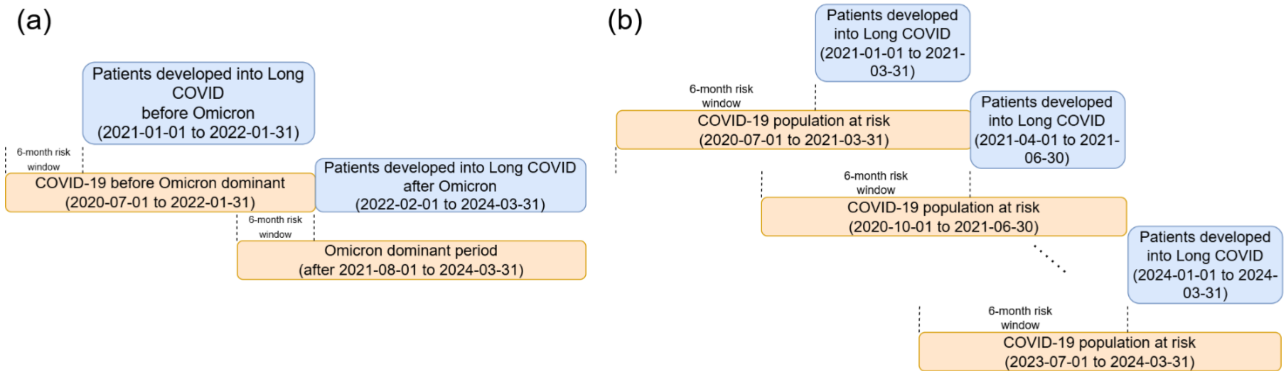

Figure S2: Dynamic incidence risk calculation. (a) The incidence of long COVID before and after the emergence of the Omicron variant in January 2022; and (b) The quarterly incidence of long COVID between 2021 and 2024.

In calculating these outcomes, we set a threshold of 180 days as a risk window to calculate dynamic incidence risk. This choice was made based on our observations from the N3C long COVID EHR and previous findings[1], where most long COVID cases (over 77%) following an acute COVID-19 infection were diagnosed within 180 days of post-infection (Figure S3 of the Supplementary Material). If a patient was not diagnosed with long COVID within this threshold, we considered them no longer at risk of developing long COVID. Based on the guidance on the International Classification of Diseases-10th Revision-Clinical Modification (ICD-10-CM), we identified acute COVID-19 using the U07.1 code to match our long-COVID definition, which relies on the B94.8 or U09.9 codes because long COVID has no corresponding biomarker tests[2]. To ensure a robust estimation of long COVID incidence during the dynamic incidence risk calculation, each time interval of interest was required to include at least 20 COVID-19 cases[3].

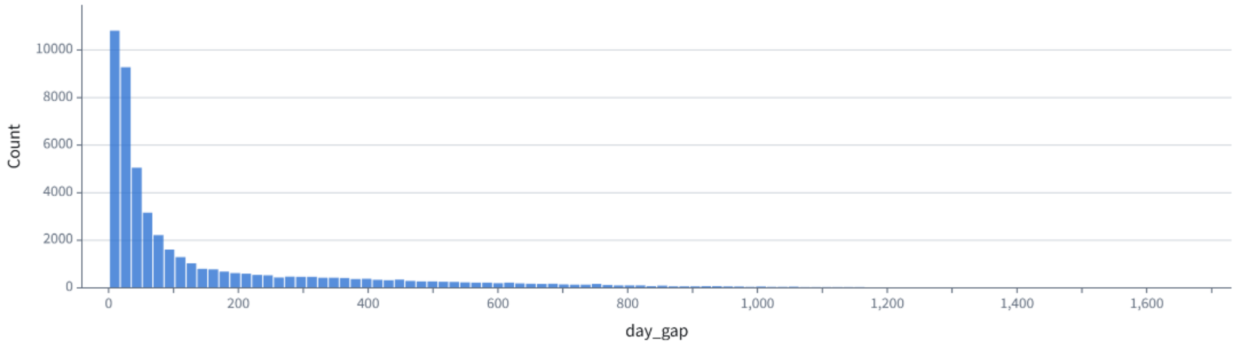

Figure S3: Summary histogram of gap days between acute COVID-19 diagnosis and long COVID diagnosis

S3 Data exploration analysis

For each county [illegible], the local Moran's I was computed as:

$$[illegible]$$

where [illegible] is the total number of U.S. counties, [illegible] is the incidence in county [illegible], [illegible] is the average incidence across counties, [illegible] is the variance of incidences, and [illegible] is the spatial weight

medRxiv preprint doi: https://doi.org/10.1101/2025.08.21.25334183; this version posted August 26, 2025. The copyright holder for this preprint (which was not certified by peer review) is the author/funder, who has granted medRxiv a license to display the preprint in perpetuity.
It is made available under a CC-BY 4.0 International license .

between counties and . The weights were defined based on semivariogram analysis results using an exponential function (**Figure** S4). Statistical significance for both methods was assessed using a Monte Carlo simulation by generating a pseudo-random distribution to determine whether the observed Moran's I and Getis is significantly different from randomness[4].

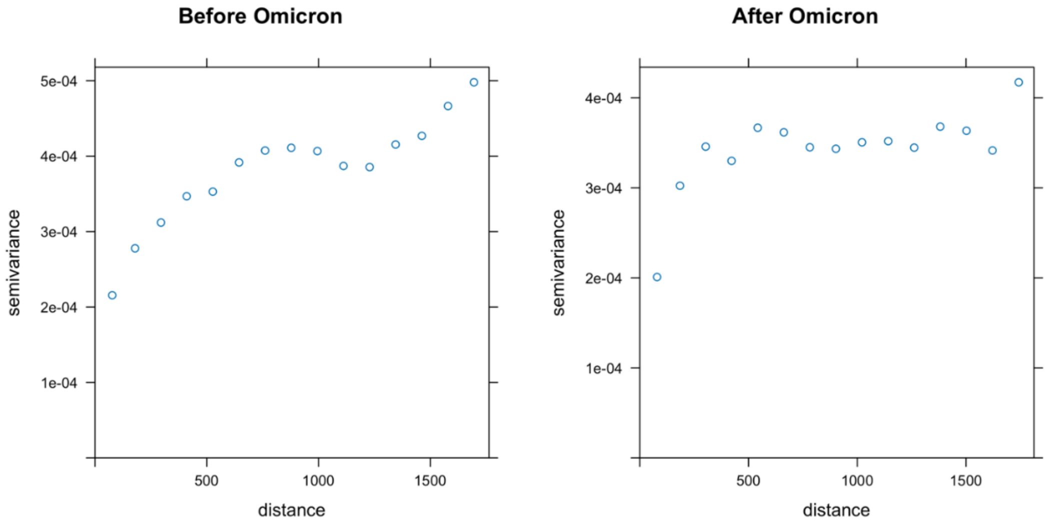

Figure S4: Semi-variogram analysis of county mean incidence before and after Omicron

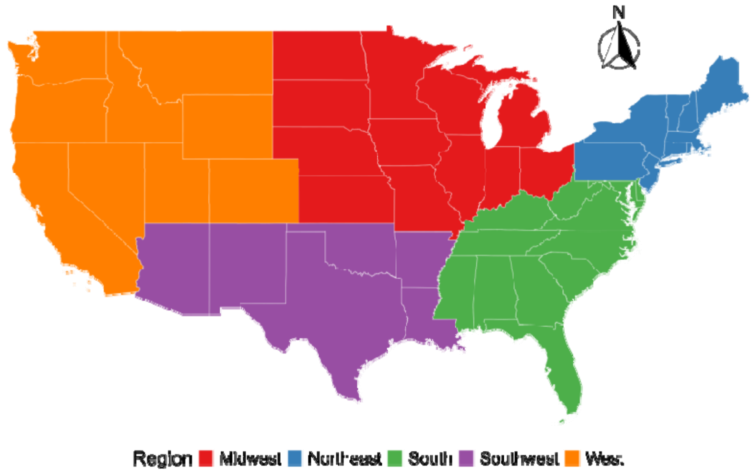

Figure S5: Five-subregion division of the United States

S4 Long COVID incidence patterns incidence before and after Omicron dominance across different subregions of the United States

Table S1. Changes in the number of counties with long COVID incidence before and after Omicron dominance across different subregions of the United States (n = 673 counties).

medRxiv preprint doi: https://doi.org/10.1101/2025.08.21.25334183; this version posted August 26, 2025. The copyright holder for this preprint (which was not certified by peer review) is the author/funder, who has granted medRxiv a license to display the preprint in perpetuity.
It is made available under a CC-BY 4.0 International license .

Region	The number of counties			
	Decreased	Increased	No Change	Increased Rate[a] (%)
Northeast	15	45	0	75.00
West	27	63	0	70.00
Midwest	72	165	0	69.62
Southwest	11	23	0	67.65
South	83	168	1	66.67
Total	208	464	1	68.95

[a]Increased Rate = Increased 100 / (Increased + Decreased + No Change).

Table S2: Average of long COVID incidence before and after Omicron across different subregions of the United States.

Region	Average incidence across counties	
	Before (%)	After (%)
Northeast	1.66	2.45
West	2.42	2.86
Midwest	2.23	2.79
Southwest	1.85	1.78
South	2.21	2.53

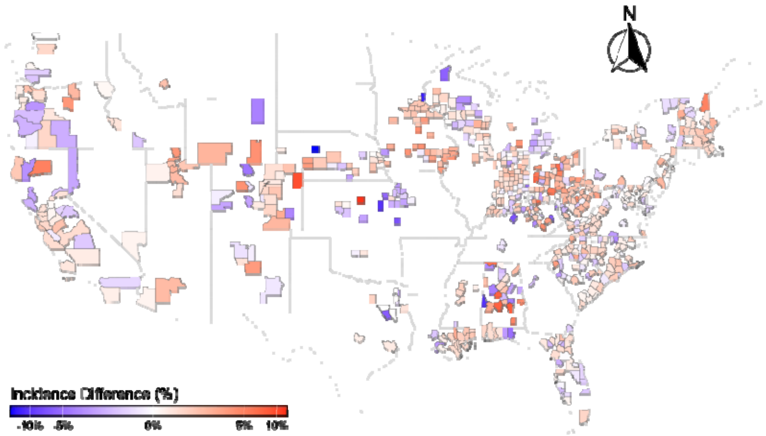

Figure S6: The map for the difference in average incidences between before and after the dominance of Omicron (i.e., January 2022).

medRxiv preprint doi: https://doi.org/10.1101/2025.08.21.25334183; this version posted August 26, 2025. The copyright holder for this preprint (which was not certified by peer review) is the author/funder, who has granted medRxiv a license to display the preprint in perpetuity.
It is made available under a CC-BY 4.0 International license .

Table S3. Regional comparison of significant local Moran's I before and after Omicron (n = 673 counties).

Region	The number (rate[a]) of counties with positive correlation		The number (rate) of counties with negative correlation	
	Before	After	Before	After
South	130 (51.6%)	128 (50.8%)	37 (14.7%)	36 (14.3%)
Northeast	26 (43.3%)	13 (21.7%)	5 (8.3%)	3 (5.0%)
Midwest	54 (22.8%)	55 (23.2%)	17 (7.2%)	30 (12.7%)
West	10 (11.1%)	29 (32.2%)	10 (11.1%)	18 (20.0%)
Southwest	0 (0.0%)	13 (38.2%)	4 (11.8%)	3 (8.8%)
Total	220 (32.7%)	238 (35.4%)	73 (10.8%)	90 (13.4%)

[a] Rate = (Number of significant counties)   100 / (Total number of counties in the region).

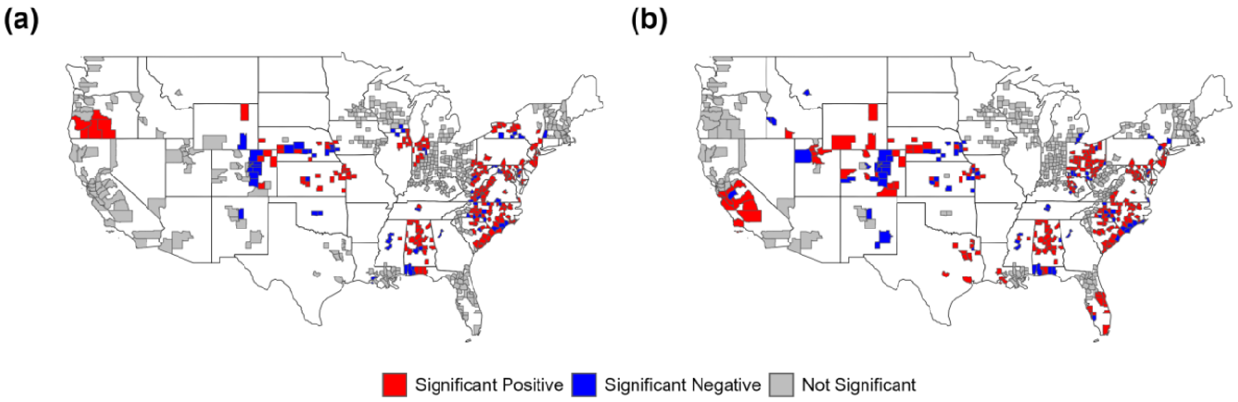

Figure S7: Spatial correlation of long COVID incidence across U.S. counties assessed using local Moran's I: (a) before and (b) after Omicron dominance.

S5 Stepwise Regression

We first adopt stepwise regression model to identify a subset of variables that best explain the variation in our study outcome, i.e., incidence of long COVID. To stable variance of incidence, we employed a logarithmic transformation for incidence (Figure S8). The results from stepwise regression were present to the following Tables S4-S5.

medRxiv preprint doi: https://doi.org/10.1101/2025.08.21.25334183; this version posted August 26, 2025. The copyright holder for this preprint (which was not certified by peer review) is the author/funder, who has granted medRxiv a license to display the preprint in perpetuity.
It is made available under a CC-BY 4.0 International license .

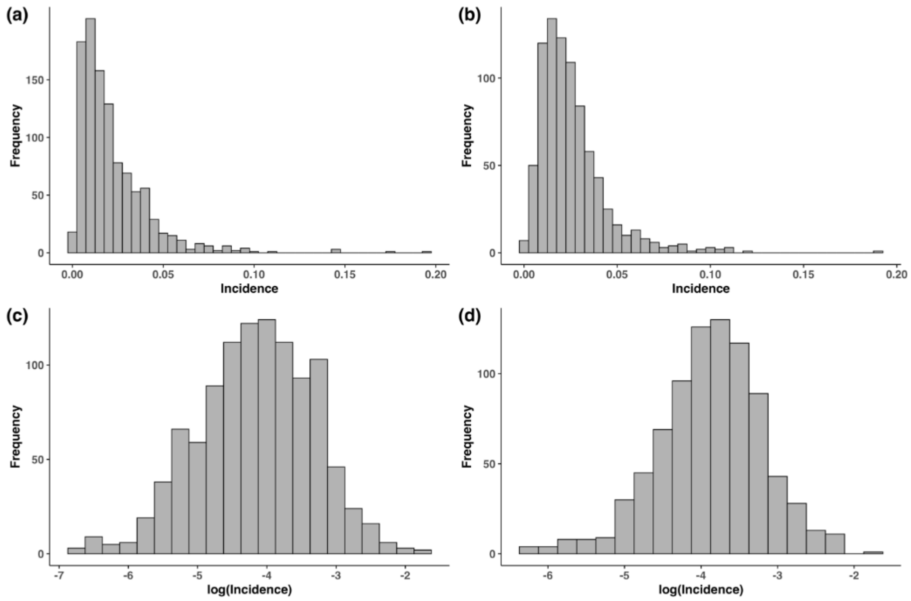

Figure S8: Distribution of county-level long COVID incidence before (a) and after (b) Omicron dominance, and the corresponding log-transformed counterparts, (c) and (d).

Table S4. Stepwise regression results for long COVID incidence before Omicron dominance.

Estimate (95% CI) p-value	Model 1	Model 2	Model 3	Model 4	Model 5
Disabled Population[a]	0.046 (0.035, 0.058) <.001*	0.037 (0.024, 0.049) <.001*	0.036 (0.023, 0.049) <.001*	0.047 (0.031, 0.062) <.001*	0.045 (0.026, 0.064) <.001*
Minority Population[b]	-0.006 (-0.009, -0.004) <.001*	-0.008 (-0.011, -0.005) <.001*	-0.007 (-0.010, -0.004) <.001*	-0.004 (-0.008, -0.001) 0.024*	-0.005 (-0.009, -0.001) 0.008*
Urban-Rural Status (Urban)	——	-0.222 (-0.331, -0.114) <.001*	-0.201 (-0.311, -0.091) <.001*	-0.203 (-0.315, -0.091) <.001*	-0.193 (-0.305, -0.080) <.001*
Housing Cost Burden	——	0.017 (0.005, 0.028) 0.004*	0.024 (0.012, 0.036) <.001*	0.020 (0.008, 0.032) 0.001*	0.020 (0.008, 0.033) 0.002*
No Vehicle Available	——	——	-0.017 (-0.028, -0.005) 0.005*	-0.015 (-0.027, -0.003) 0.013*	-0.013 (-0.025, -0.001) 0.029*
First Vaccination Rate	——	——	-0.004 (-0.008, -0.001) 0.016*	-0.005 (-0.009, -0.001) 0.007*	-0.006 (-0.009, -0.002) 0.004*
No High School Diploma	——	——	——	-0.018 (-0.032, -0.003) 0.015*	-0.029 (-0.046, -0.011) 0.001*
Group Quarters	——	——	——	0.015 (0.001, 0.030) 0.036*	0.015 (0.000, 0.030) 0.044*

medRxiv preprint doi: https://doi.org/10.1101/2025.08.21.25334183; this version posted August 26, 2025. The copyright holder for this preprint (which was not certified by peer review) is the author/funder, who has granted medRxiv a license to display the preprint in perpetuity.
It is made available under a CC-BY 4.0 International license .

Crowded Housing	——	——	——	——	0.033<br>(-0.008, 0.075)<br>0.117
Mobile Homes	——	——	——	——	0.006<br>(-0.002, 0.014)<br>0.122

[a] Disability refers to the proportion of the civilian noninstitutionalized population with any disability; [b] The minority status refers to the percentage of the population that is not non-Hispanic White.

Table S5. Stepwise regression results for long COVID incidence after Omicron dominance.

OR<br>(95%CI)<br>p-value	Model 1	Model 2	Model 3	Model 4	Model 5
Minority Population	-0.012<br>(-0.015, -0.010)<br><.001*	-0.010<br>(-0.012, -0.007)<br><.001*	-0.009<br>(-0.012, -0.006)<br><.001*	-0.009<br>(-0.013, -0.006)<br><.001*	-0.010<br>(-0.013, -0.006)<br><.001*
Below 150% Poverty Level	0.015<br>(0.009, 0.021)<br><.001*	0.018<br>(0.010, 0.027)<br><.001*	0.012<br>(0.002, 0.023)<br>0.020*	0.013<br>(0.002, 0.023)<br>0.019*	0.014<br>(0.003, 0.025)<br>0.011*
Urban-Rural Status (Urban)	——	-0.160<br>(-0.264, -0.056)<br>0.003*	-0.187<br>(-0.293, -0.080)<br><.001*	-0.173<br>(-0.280, -0.067)<br>0.001*	-0.141<br>(-0.249, -0.033)<br>0.010*
No High School Diploma	——	-0.019<br>(-0.032, -0.006)<br>0.003*	-0.023<br>(-0.036, -0.010)<br><.001*	-0.039<br>(-0.055, -0.023)<br><.001*	-0.047<br>(-0.064, -0.030)<br><.001*
Population Age 65+	——	——	-0.017<br>(-0.030, -0.005)<br>0.008*	-0.017<br>(-0.029, -0.004)<br>0.011*	-0.019<br>(-0.032, -0.006)<br>0.005*
Disabled Population[b]	——	——	0.021<br>(0.003, 0.040)<br>0.023*	0.031<br>(0.012, 0.051)<br>0.002*	0.031<br>(0.011, 0.050)<br>0.002*
Limited English	——	——	——	0.039<br>(0.011, 0.068)<br>0.007*	0.056<br>(0.026, 0.087)<br><.001*
First Vaccination Rate	——	——	——	-0.005<br>(-0.009, -0.001)<br>0.010*	-0.004<br>(-0.008, 0.000)<br>0.052
Group Quarters	——	——	——	——	0.016<br>(0.002, 0.030)<br>0.025*
Multi-Unit Housing	——	——	——	——	-0.012<br>(-0.021, -0.003)<br>0.009*

medRxiv preprint doi: https://doi.org/10.1101/2025.08.21.25334183; this version posted August 26, 2025. The copyright holder for this preprint (which was not certified by peer review) is the author/funder, who has granted medRxiv a license to display the preprint in perpetuity.
It is made available under a CC-BY 4.0 International license .

S6 Spatial random effect models

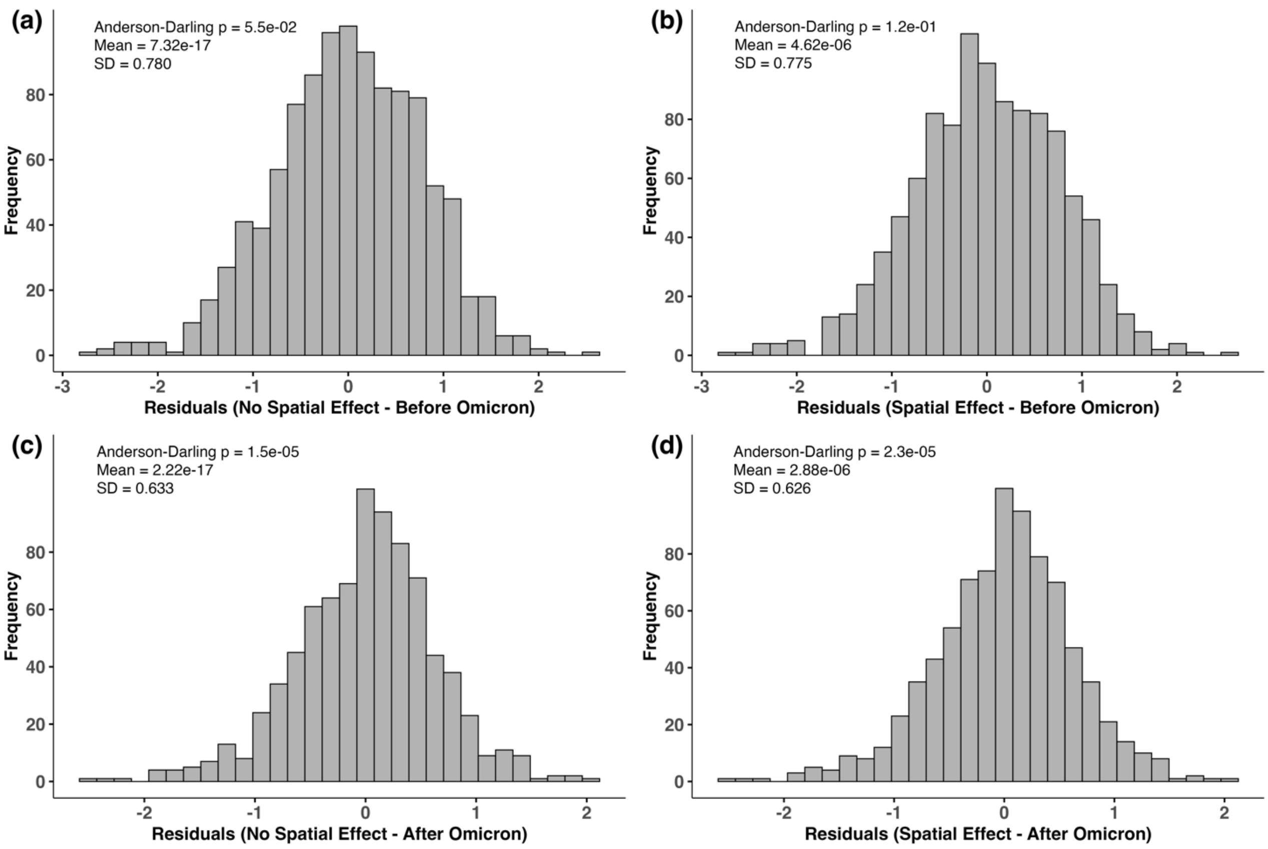

Figure S9: Comparison of residual distributions before and after incorporating spatial random effects in models fitted before and after Omicron dominance: (a) No spatial effect – before Omicron; (b) Spatial effect – before Omicron; (c) No spatial effect – after Omicron; (d) Spatial effect – after Omicron.

medRxiv preprint doi: https://doi.org/10.1101/2025.08.21.25334183; this version posted August 26, 2025. The copyright holder for this preprint (which was not certified by peer review) is the author/funder, who has granted medRxiv a license to display the preprint in perpetuity.
It is made available under a CC-BY 4.0 International license .

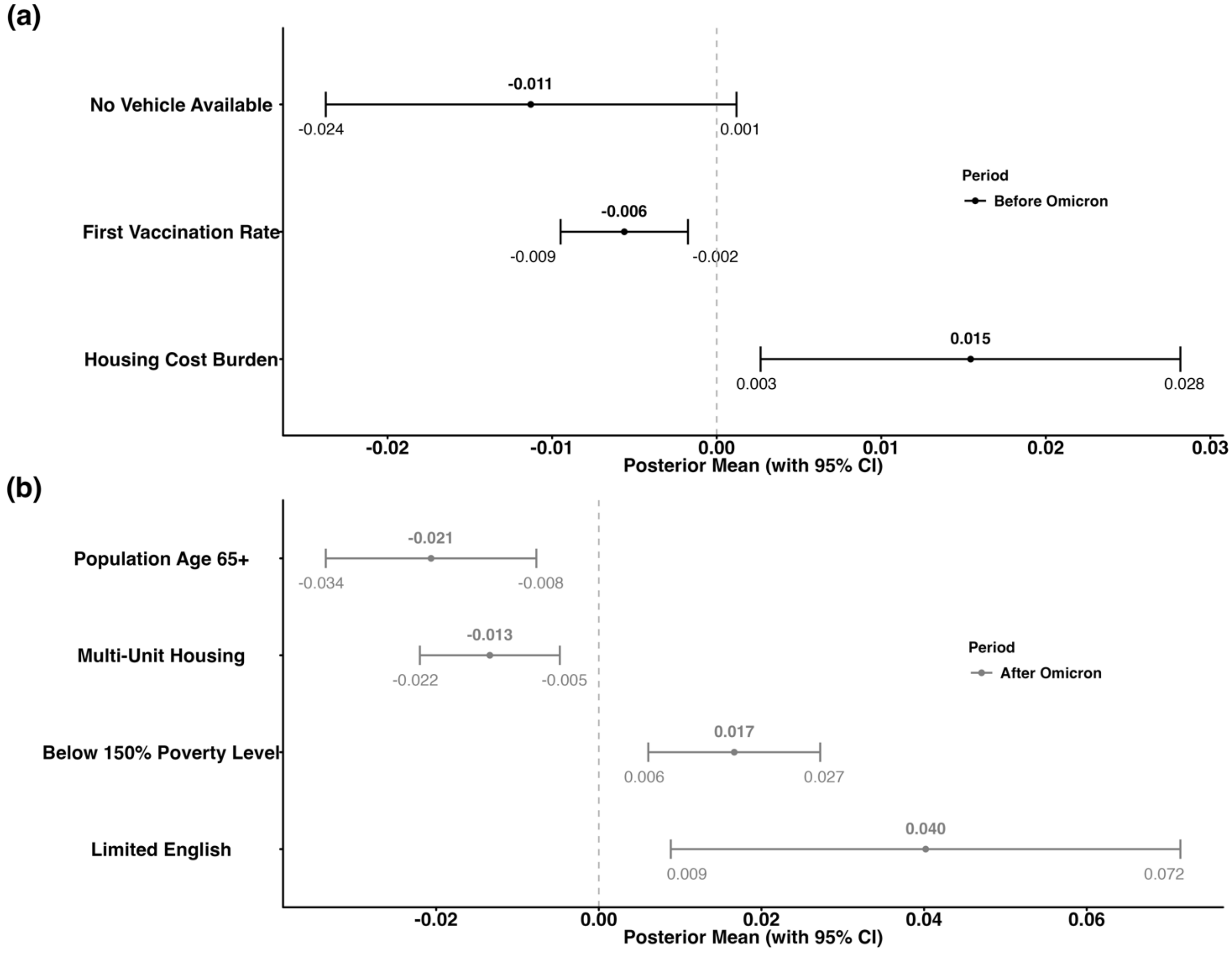

Figure S10: Spatial random effect models yielded posterior estimates (95% credible intervals (CIs)) of variables uniquely selected before (a) and after (b) Omicron dominance.

Table S6. Regional differences in long COVID incidence by social vulnerability factors thresholds

Variable	Region	Average incidence across counties (%)			
		Level ≤ Threshold	Level > Threshold	Difference	p value
Disability Status Group (threshold: 10%)	Midwest	0.87	1.62	0.75	<0.0001
	Northeast	0.75	1.45	0.7	<0.0001
	South	0.61	1.46	0.85	<0.0001
	Southwest	1.33	1.13	-0.2	0.0687
	West	1.32	1.99	0.67	0.0007
First Vaccine Rate (threshold: 60%)	Midwest	1.85	1.27	-0.58	<0.0001
	Northeast	1.38	1.33	-0.05	0.0254
	South	1.75	1.2	-0.55	<0.0001
	Southwest	1.26	0.99	-0.27	0.0994
	West	2.99	1.58	-1.41	<0.0001

medRxiv preprint doi: https://doi.org/10.1101/2025.08.21.25334183; this version posted August 26, 2025. The copyright holder for this preprint (which was not certified by peer review) is the author/funder, who has granted medRxiv a license to display the preprint in perpetuity.
It is made available under a CC-BY 4.0 International license .

Group Quarters Population (threshold: 3%)	Midwest	1.48	1.71	0.23	0.0166
	Northeast	1.27	1.41	0.14	0.0299
	South	1.34	1.58	0.24	<0.0001
	Southwest	1.11	1.25	0.14	0.1059
	West	1.89	1.82	-0.07	0.9177
Minority Population (threshold: 20%)	Midwest	1.61	1.37	-0.24	0.0006
	Northeast	1.6	0.91	-0.69	<0.0001
	South	1.64	1.21	-0.43	<0.0001
	Southwest	1.28	1.13	-0.15	0.1022
	West	2.22	1.59	-0.63	<0.0001
Population Below 150% Poverty (threshold: 12%)	Midwest	0.98	1.61	0.63	<0.0001
	Northeast	0.89	1.43	0.54	0.0003
	South	0.7	1.46	0.76	<0.0001
	Southwest	1.69	1.13	-0.56	0.0219
	West	1.59	1.9	0.31	0.1979
Rural-Urban Category (Urban: Rural)	Midwest	1.47	1.73	0.26	<0.0001
	Northeast	1.2	1.68	0.48	<0.0001
	South	1.17	1.98	0.81	<0.0001
	Southwest	1.03	1.65	0.62	<0.0001
	West	1.6	2.76	1.16	<0.0001

Reference

1. Bowe B, Xie Y, Al-Aly Z. Postacute sequelae of COVID-19 at 2 years. *Nature medicine.* 2023;29(9):2347-2357.
2. ICD10data.com. 2024 ICD-10-CM diagnosis code U09. 2024; https://www.icd10data.com/ICD10CM/Codes/U00-U85/U00-U49/U09-/U09.9. Accessed January 1, 2025.
3. Parker J, Talih M, Malec DJ, et al. National Center for Health Statistics data presentation standards for proportions. 2017.
4. Anselin L. Local indicators of spatial association—LISA. *Geographical analysis.* 1995;27(2):93-115.
